# Homologous recombination repair and breast cancer gene testing in prostate cancer: expert perspectives and practical guidance on best practice from sample acquisition to biomarker-informed clinical decision-making

**DOI:** 10.3389/fonc.2026.1874947

**Published:** 2026-07-01

**Authors:** Eva Compérat, Gunhild von Amsberg, Lynda Corrigan, Stephen P. Finn, Glen Kristiansen, Angelo Minucci, David Olmos, Guilhem Roubaud

**Affiliations:** 1 Department of Pathology, The Medical University of Vienna, Vienna, Austria; 2 University Cancer Center Hamburg and Martini-Klinik, University Hospital Hamburg Eppendorf, Hamburg, Germany; 3 Department of Medical Oncology, Tallaght University Hospital, Dublin, Ireland; 4 Cancer Molecular Diagnostics, Trinity St. James's Cancer Institute, Dublin, Ireland; 5 Institute of Pathology, University Hospital Bonn, Bonn, Germany; 6 Departmental Unit of Molecular and Genomic Diagnostics, Fondazione Policlinico Gemelli Istituto di Ricovero e Cura a Carattere Scientifico (IRCCS), Rome, Italy; 7 Instituto de Investigación Hospital 12 de Octubre, Hospital Universitario 12 de Octubre, Madrid, Spain; 8 Department of Medical Oncology Institut Bergonié Bordeaux, Bordeaux, France

**Keywords:** BRCA mutations, clinical decision-making, germline and somatic, HRR, liquid biopsy, molecular diagnostics, PARP inhibitors, prostate cancer

## Abstract

The prostate cancer (PCa) treatment landscape, particularly metastatic PCa, is rapidly evolving, with an increase in personalised treatment approaches and therapy selection being guided by tumour genomics. However, PCa remains the third leading cause of death among male-specific cancers in the European Union. Nearly a quarter of patients with metastatic PCa harbour homologous recombination repair (HRR) pathway mutations, with approximately 12% harbouring alterations in the *BRCA*1 or *BRCA*2 genes. These genomic alterations are associated with poorer prognosis and increased sensitivity to targeted therapies, such as poly(adenosine diphosphate-ribose) polymerase inhibitors. There is an urgent need to implement HRR and *BRCA* testing early in the treatment pathway. Despite treatment advances, early testing is not routinely implemented in clinical practice. Barriers include unequal testing access, a lack of clarity on who, when, and how to test, and how to interpret test results accurately. These may prevent eligible patients from receiving treatment early. To address this, we combine our clinical experience with the current literature to support the wider adoption of HRR/*BRCA* testing by providing practical recommendations for implementing best practices along the PCa molecular testing pathway. Our recommendations aim to help identify suitable patients for early testing and guide how to obtain, interpret, and utilise molecular test results in clinical decision-making.

## 1 Introduction

Prostate cancer (PCa) remains the most diagnosed cancer among men and the third leading cause of death among all male-specific cancers in the European Union, with 1.4 million new cases reported worldwide in 2020 (1, 2). Approximately one in four patients with metastatic PCa have homologous recombination repair (HRR) pathway gene mutations, >12% of which include breast cancer genes 1 and 2 (*BRCA*1/*BRCA*2) alterations (3), which have key implications for prognosis and treatment selection. The prompt and accurate testing of these mutations may help identify appropriate therapy earlier for specific patients, while also providing prognostic information and the need for cascade testing of family members during positive germline findings.

PCa management has progressed from a disease-stage and histology-based approach to a more personalised approach (4). Many patients with *BRCA*-mutated metastatic PCa who may be eligible for targeted therapies may not be identified promptly due to inconsistent testing practices or long turnaround times between sample collection and test reporting.

Treatment options vary based on clinicopathological and molecular features. PCa patients with *BRCA* mutations have worse prognosis (3, 5). Although there is growing evidence supporting molecular testing (6–8), guidelines lack practical recommendations, and barriers persist regarding who, when, and how to test. There are additional complexities in interpreting next-generation sequencing (NGS) test results for clinical decision-making. Optimising testing capabilities may enable more precise diagnoses and better patient outcomes.

**Figure 1:**
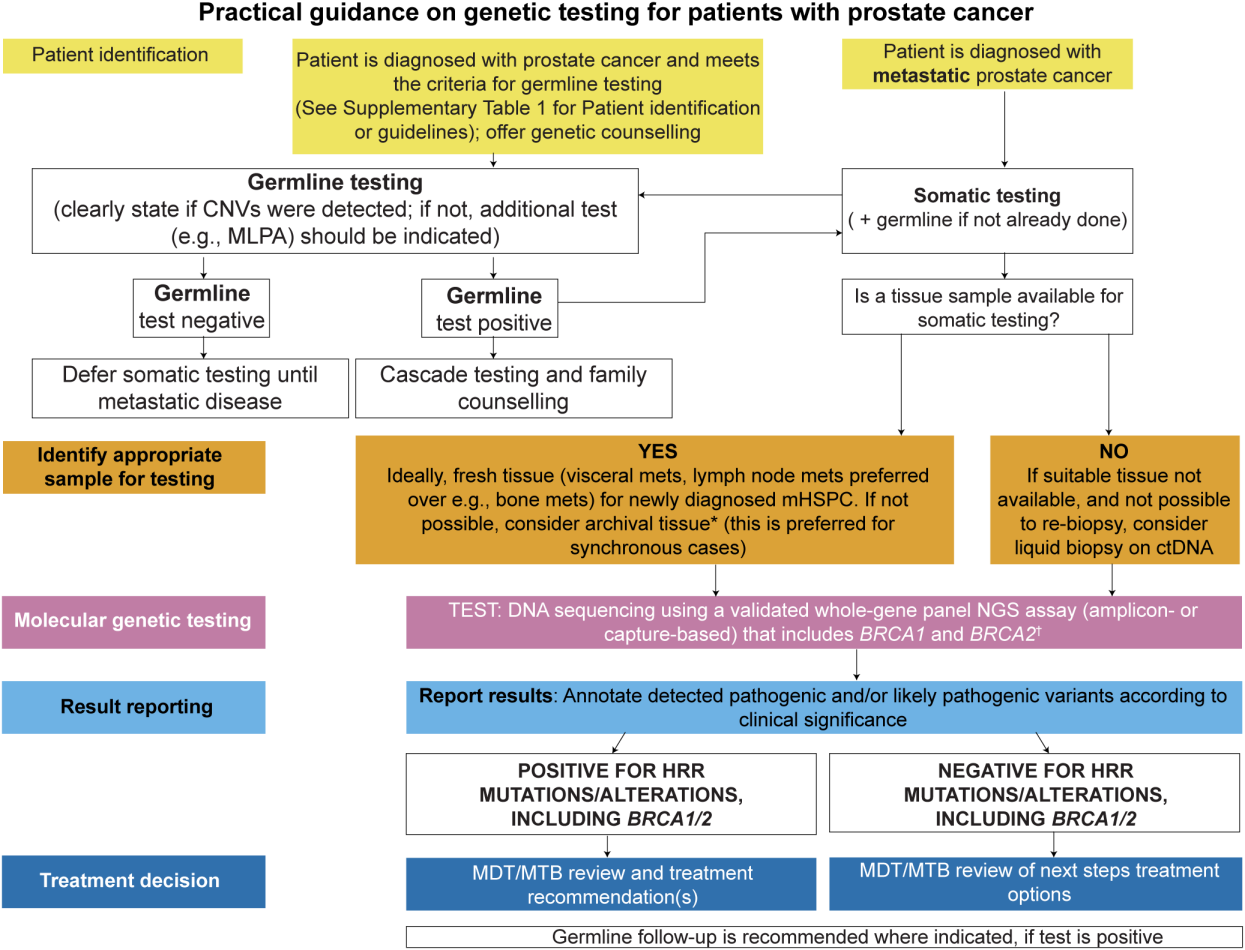
Practical guidance on genetic testing for patients with prostate cancer. *Target time from sample collection or retrieval of archival sample to report should ideally be 2–3 weeks. ^†^HRR genes to be assessed depend on country-specific licence indications and reimbursement approvals. *BRCA1/2*, breast cancer genes 1/2; CNVs, copy number variations; ctDNA, circulating tumour DNA; HRR, homologous recombination repair; MDT, multidisciplinary team; mets, metastases; mHSPC, metastatic hormone-sensitive prostate cancer; MLPA, multiplex ligation-dependent probe amplification; MTB, molecular tumour board; NGS, next-generation sequencing.

We aim to highlight the unmet need for appropriate genetic testing and educate on the key steps along the molecular testing pathway in PCa. Based on the clinical experience of a multidisciplinary group of healthcare professionals (HCPs), covering expertise in oncology, pathology, and molecular biology and the current literature, we provide practical perspectives and recommendations for implementing best practices in HRR/*BRCA* testing for PCa, from identifying suitable patients for testing, to obtaining, interpreting, and utilising molecular test results in clinical decision-making. **Figure 1** provides practical guidance on PCa genetic testing.

### 1.1 Equity and access: why testing should extend beyond academic centres

Access to PCa screening varies across Europe. *BRCA* testing is reimbursed in some countries (9), often because some countries or regions have more comprehensive local guidelines than others (10, 11) and/or limited funding, especially in public health systems. There is a need to implement clear, replicable testing pathways for urologists, oncologists, pathologists, and other HCPs. Testing should extend beyond genomic specialists and academic centres because improving equity and access enables the identification of eligible patients for specific treatments.

## 2 Why test early? The role of HRR/*BRCA* alterations in PCa

### 2.1 Genomic alterations and survival outcomes in PCa

The prevalence of HRR/*BRCA* pathogenic variants is comparable in patients with metastatic hormone-sensitive prostate cancer (mHSPC) and metastatic castration-resistant prostate cancer (mCRPC), showing the need for testing at the time of metastatic diagnosis (3, 12). In mHSPC, 29% of patients harbour mono- or bi-allelic HRR alterations, similar to 31% in mCRPC; *BRCA1/2* alterations occur in approximately 12% of mHSPC patients and 13% with mCRPC, including homozygous or heterozygous deletions (3, 12, 13), highlighting the prevalence of clinically actionable mutations across disease states. Combined with other data from matched primary and mCRPC biopsies, this supports that *BRCA* mutations are early truncal events, supporting early testing (14).

HRR/*BRCA* somatic or germline alterations are associated with poor prognosis (3, 5, 15, 16). A study of 556 conventionally treated mHSPC patients showed that HRR alterations, especially *BRCA* alterations, were associated with significantly worse prognosis, irrespective of disease volume or treatment regimen (3, 12).

Identifying *BRCA1/2* mutations in PCa patients has direct implications for eligibility for poly(adenosine diphosphate-ribose) polymerase inhibitors (PARPis) in metastatic PCa (6, 15, 17–21) and predicts treatment response (3, 5, 15, 16). PARPis provide a more tailored treatment approach by inducing synthetic lethality (22). The 2026 European Association of Urology (EAU) guidelines recommend somatic or germline testing for HRR aberrations in patients with metastatic PCa, as mHSPC patients may qualify for treatment with niraparib plus abiraterone and prednisone, in combination with androgen deprivation therapy. Niraparib is the first PARPi regimen approved by the European Medicines Agency for *BRCA1*/*2*-mutated mHSPC patients, emphasising the importance of implementing early HRR/*BRCA* testing in routine clinical practice (6, 23).

### 2.2 Broader implications of early testing

Beyond the therapeutic benefits of identifying patients with HRR/*BRCA* alterations, early testing can assess hereditary cancer risk. *BRCA* mutations can be somatic or germline, and both can guide treatment decisions (24). The ideal testing strategy should detect somatic and germline mutations, capturing the full spectrum of HRR alterations early. NGS performed on tumour tissue or liquid biopsies can identify somatic alterations and suggest potential germline variants, but definitive confirmation of germline mutations requires blood or saliva DNA testing.

By identifying HRR/*BRCA* mutations, HCPs can better tailor treatment decisions to the underlying tumour biology and provide opportunities for genetic counselling and cascade testing.

## 3 Practical guidance to patient identification and testing

Accurately identifying the right patients to test at the right time (**Figure 1**) is crucial for treatment eligibility and consistently implementing molecular testing across institutions.

### 3.1 Who and when to test

Several clinical guidelines have been developed for PCa management, including recommendations for testing (**Supplementary Table 1**). The 2026 National Comprehensive Cancer Network Prostate Cancer Guidelines recommend that patients with a family history and/or high-risk localised PCa undergo germline testing, and patients with metastatic PCa undergo somatic testing for HRR alterations (7). They also recommend considering germline testing in patients who are likely to be affected by their PCa treatment and clinical trial options, for managing the risk of other cancers, and/or for the potential risk of cancer in family members (7). European guidelines, including the European Society of Medical Oncology and the EAU guidelines, support early testing in PCa (6, 8). The EAU guidelines recommend somatic or germline HRR testing in mHSPC patients, reinforcing the importance of early testing at metastatic diagnosis (6).

Considering this and the current PCa landscape, we recommend that standardised processes should be in place across institutions to ensure patients are tested early, ideally at mHSPC diagnosis.

Testing at mCRPC progression often causes practical challenges (e.g., insufficient tissue for testing, sample degradation, or delays in obtaining results), which affect treatment timing. Waiting until disease progression is scientifically unnecessary, as HRR alterations are detectable in both primary and metastatic biopsies (18, 25, 26). However, estimating true HRR prevalence remains challenging, given that only a small proportion of patients with PCa currently undergo molecular testing in routine clinical practice, particularly in the metastatic setting (26, 27). Benefits of early testing include:

- Increased access to younger, better-preserved archival tissue.
- Opportunity to obtain fresh/recent metastatic tissue, enabling high-quality DNA/RNA for testing.
- Treatment decisions are more timely, e.g., at mHSPC diagnosis, because patients with HRR alterations (especially *BRCA1*/*2*) may develop castration resistance earlier and have a poorer prognosis (3, 5, 15).

Solutions to minimise testing delays include requesting tissue samples at diagnosis or integrating molecular testing into routine workflows, ideally through reflex testing protocols (i.e., performing additional tests when particular criteria are met, without waiting for additional testing requests).

### 3.2 Testing methodology: somatic vs germline testing

Currently, somatic HRR/*BRCA* mutation testing is mostly conducted at the mCRPC stage; however, dual testing (e.g., germline and somatic) at the mHSPC stage is preferred to capture the full spectrum of HRR alterations early.

Both tests offer key clinical insights:

- **Somatic testing** identifies acquired and inherited genomic alterations (15). However, it cannot distinguish between germline or somatic mutations; somatic-only testing may miss germline alterations (28, 29). A positive somatic test for HRR/*BRCA* genes should be followed up with a germline test (30).
- **Germline testing** identifies familial cancer risk and inherited mutations and confirms somatic test findings (30).

Despite the importance of HRR/*BRCA* testing in PCa, challenges remain for the broader adoption of routine testing (31). A study showed that among 39% of eligible PCa patients, only 11% completed testing (32).

## 4 How to test in practice: from biopsy to clinical decision-making

Knowing how to test can ensure the results are accurate and clinically useful. HRR/*BRCA* testing availability can vary depending on local reimbursement and health system infrastructure. Testing coverage should be verified in line with local and national regulations.

We provide a stepwise approach to testing, reflecting the current literature, international guidelines, and our experience.

### 4.1 Tissue acquisition, fixation, and storage

Successful HRR/*BRCA* testing begins with acquisition, fixation, and high-quality tissue storage (33). Tissue samples have several advantages over liquid biopsies: block selection, marking the tumour, and cutting slices of unstained material (33). Tissue samples can be obtained via radical prostatectomy. Metastatic tissue is preferred, but adequate tissue is often unavailable. Although molecular testing is possible from fresh and archived tissue, tissue from prostate biopsies may be limited by insufficient material. While recently acquired tissue is ideal, obtaining fresh/recent tissue at the time of progression can be challenging in bone-predominant metastatic disease and carries biopsy-associated risks. Where possible, tissue samples for molecular testing should be prioritised after metastatic diagnosis, using formalin-fixed paraffin-embedded (FFPE) tissue, after sufficient tissue has been obtained for histopathological confirmation.

As a result, HCPs often rely on archival tissue, which can be problematic because it is often too old/degraded or processed under suboptimal conditions. Logistical challenges (i.e., delays in retrieving and accessing archival samples) could contribute to these issues and significantly increase test result turnaround times (34). Regardless of where the tissue is obtained from, pathologists require a minimum amount of tumour material. The molecular integrity and test results of tissue samples can be impacted by factors like sample age and cold ischaemia (**Supplementary Figure 1**) (33, 35, 36).

Recently acquired tissue is preferred for analysis, with visceral or lymph node metastases prioritised over bone metastases; the testing success rate for bone and lymph node is 42% and 74%, respectively (37). An archival tissue sample (ideally <5 years) may provide successful results.

For newly diagnosed mHSPC, it is preferable to use fresh/recently acquired tissue from a core needle biopsy for genomic or HRR/*BRCA* testing, as it provides the most reliable DNA for molecular analysis. In synchronous cases, re-biopsy is not necessarily required immediately: archival tissue can be used initially to avoid unnecessary invasive biopsies. If fresh tissue is unavailable or patients do not consent to a fresh biopsy collection, archival tissue may be used. When fresh/archival tissue is unsuitable and re-biopsy is impossible, liquid biopsy using circulating tumour DNA (ctDNA) should be considered.

### 4.2 Pre-analytics on fixed tissue

After tissue acquisition and fixation, pre-analytics to extract high-quality RNA/DNA is next.

#### 4.2.1 Appropriate tissue selection

Choosing the appropriate tissue block depends on various factors, including sample size, tumour cellularity, sample age, and collection method (26, 35). Where feasible, blocks with high tumour content (≥5,000 tumour cells) should be prioritised by pathologists to ensure sufficient DNA quantity and to reduce repeat testing. Samples that have been poorly preserved or degraded should be avoided. The American Society of Clinical Oncology and the College of American Pathologists recommend selecting the tissue sample with the highest volume and highest Gleason pattern (38).

#### 4.2.2 Macrodissection and quantification of tumour cell content

Macrodissection and algorithms could enrich tumour fraction and increase HRR/*BRCA* testing sensitivity (39), as most NGS assays require a minimum tumour cell content threshold (40). This requires carefully isolating tumour-enriched areas on tissue slides to increase the chance of extracting nucleic acid from tumour cells.

To accurately guide dissection localisation, the tumour should be clearly marked by a pathologist on haematoxylin and eosin (H&E)-stained slides. At least 5,000 tumour cells or tumour content of more than 30% is ideal for reliable results. Going below these can increase the risk of false negatives or assay failure and hinder the detection of low-allele-frequency somatic mutations (35). Four to six 5 µm-thick tissue sections are best for DNA extraction.

Most patients with metastatic PCa have bone metastases (26, 41). Collecting samples from bone-predominant metastatic sites is challenging for patients and HCPs due to morbidity and procedure invasiveness (35). Processing bone biopsy samples often requires acid-based decalcification, degrading DNA quality (35). Hence, to test bone metastatic tumours and preserve DNA quality, ethylenediaminetetraacetic acid (EDTA)-based decalcification is preferred over harsher decalcification processes (35). Early communication with the pathology team ensures proper sample processing when collecting bone metastases.

#### 4.2.3 Nucleic acid extraction, amplification, and library preparation

High-quality DNA or RNA extraction is another crucial step in the testing pathway. Generally, nucleic acid from fresh/recently acquired FFPE samples is of good quality; however, quality declines over time due to degradation and chemical modification (35).

Laboratories must use validated extraction protocols designed for FFPE samples to ensure the proper quality and integrity of nucleic acids. Otherwise, downstream sequencing errors and post-library test failures can occur. To minimise this, pre-analytical quality control of samples should be performed (35), to allow the early detection of degradation or low-yield samples and prevent wasted sequencing runs.

After DNA extraction, library preparation is performed, using amplification-based capture or hybridisation-based capture, before pooled enrichment. Importantly, these two approaches differ in their ability to detect copy number alterations. Hybridisation-based capture methods are generally preferred over amplification-based capture for detecting lower-frequency variants, offering better coverage uniformity and homozygous deletions detection, especially *BRCA2*/other HRR alterations (42–44). To reduce the risk of post-library test failures, it is important to quantify double-stranded DNA yield and confirm DNA amplification possibility (45).

Validated NGS assays that cover all the clinically relevant coding regions of *BRCA1/2* and other HRR genes of interest should be used, depending on local reimbursement rules. **Table 1** shows the dos and don’ts of the steps in the testing pathway.

**Table 1:** The dos and don’ts of each step in the molecular diagnostic testing pathway for metastatic prostate cancer.

Step	Do	Don’t
Tissue sample acquisition	Request a recently acquired tissue biopsy, where possible. If recently acquired tissue is not available, request archival tissue, ideally no more than 5 years old. Have one biopsy per container/block.	Have more than three samples per container.
Liquid biopsy	Use two tubes specific to liquid biopsy. Avoid genomic DNA release from lysing leukocytes to avoid false-negative results. Probe within 1 hour if using EDTA-3K tubes. If immediate processing is not possible, store at 4 °C for up to 24 hours.	Use poorly preserved tissue samples, damaging cellular components. Freeze, share, or store at 4 °C for longer than 24 hours for DNA stabilisation before plasma separation.
Fixation	Use 7.5% or 10% neutral phosphate-buffered formalin. Fixation time of at least 6 hours.	Overfixate or underfixate.
Storage	Have slide archives and tissue block archives so that if slides are lost, blocks can always be used. Ensure blocks are arranged in the correct order to avoid delays or errors. Store FFPE samples at room temperature (18–25 °C).	Store in humid conditions.
Tissue sample selection	Use blocks with ≥30% tumour content and ≥5,000 tumour cells. Use freshly cut slices for better results. Mark the tumour on the H&E-stained slide for macrodissection to enrich tumour fraction if tumour content is low.	Use samples with extensive necrosis. Use samples with low tumour cell count. Use too few tumour cells for DNA/RNA extraction.
Decalcification of bone metastases	If required, use EDTA-based decalcification.	Use poorly preserved tissue samples, damaging cellular components. Use acidic decalcification

DNA, deoxyribonucleic acid; EDTA, ethylenediaminetetraacetic acid; FFPE, formalin-fixed paraffin-embedded; H&E, haematoxylin and eosin; RNA, ribonucleic acid.

### 4.3 Liquid biopsy: an option if tissue testing is impossible

Liquid biopsy biomarkers (e.g., ctDNA *BRCA* and *ATM* alterations) have shown promise for guiding treatment selection (e.g., PARPis) in metastatic PCa (46, 47); however, liquid biopsy testing still requires prospective validation in PCa. Although using liquid biopsy is not the ideal first-line approach for HRR/*BRCA* testing in PCa (**Figure 1**, **Supplementary Figure 2**), it can be useful under certain circumstances:

- When tumour tissue is inadequate/unavailable.
- To provide a more accurate view of the tumour’s mutational status at the time of sample collection (48).
- When a non-invasive route is preferred/safer (48).

Key parameters to consider when reducing the risk of missing patients with actionable mutations using liquid biopsy testing include tumour fraction, tumour mutational burden, microsatellite status, alterations and variant allele frequency (VAF), and alteration pathogenicity.

There are several limitations associated with liquid biopsy (see **Supplementary Figure 3**) (49–53). It should be used with caution, as routine liquid biopsy testing in PCa still requires prospective trial validation.

### 4.4 Post-analytics: reporting and clinical decision-making

After NGS testing, the last step is to produce results that are clear and correctly interpreted for clinical decision-making. Depending on the country, clinical decision-making requires a multidisciplinary team (MDT) or a molecular tumour board, comprising oncologists, urologists, radiologists, pathologists, and molecular geneticists. When HRR mutations are detected through germline testing, patients should be referred to a clinical geneticist or genetic counsellor to explain the molecular test results and to offer genetic risk assessment to patients’ families (54, 55).

In addition to ensuring that pathologists are included in molecular tumour boards to provide the necessary expertise when reviewing results (56), test reports should indicate their clinical relevance. Liquid biopsy results must be interpreted with caution, given their limitations. It should be acknowledged that HRR testing results may vary across laboratories due to differences in NGS platforms, assay designs, bioinformatic pipelines, and variant interpretation criteria, which presents a real-world challenge for result standardisation and cross-institutional comparability.

Including critical data like allele frequency in testing reports can provide better insight into clinical implications and treatment eligibility. Clinicians may be presented with reports that lack clear interpretation or relevant treatment context. A solution is to ensure that genetic reports include clear, actionable summaries of clinical significance (**Supplementary Table 2**).

We identified common pitfalls and several recurring issues that lead to failed or inconclusive tests, including limited communication across specialities, incomplete lab request forms, unclear result reporting, and unclear clinical context, which may compromise test results. Our solutions to minimise failure rates and to avoid common pitfalls are shown in **Supplementary Table 2**.

## 5 Discussion and future perspectives

HRR/*BRCA* testing is crucial in PCa management, with direct implications for therapy selection and prognosis. Early identification of HRR/*BRCA* mutations can help clinicians understand the underlying tumour biology and personalise treatment for patients more likely to benefit from PARPis.

Our proposed practical approaches to implementing HRR/*BRCA* testing in everyday clinical practice could increase testing adoption in clinical settings, especially outside academic centres, and may lead to increased reflex testing. Increasing adoption requires streamlined workflows and greater HCP awareness. That is, educating clinicians and streamlining access to testing are key to scaling up molecular testing and ensuring broader implementation in routine clinical practice. Ideally, an optimised testing strategy involves starting with fresh/recent tissue if available, avoiding common pitfalls along the testing pathway, and reporting clear test results to MDTs.

Implementing HRR/*BRCA* testing may require navigating the local reimbursement criteria, given the current significant variation in test reimbursement across countries. This further emphasises the need for national frameworks for equal access to testing.

Emerging data on PARPi resistance mechanisms are relevant to this evolving landscape. HRR-related genomic alterations, including reversion mutations in *BRCA1/2*, may emerge following PARPi exposure, underscoring the dynamic nature of tumour genomics in treated patients (57). In this context, liquid biopsy offers a potentially valuable tool for monitoring and detecting resistance-associated alterations in a minimally invasive manner, though prospective validation in PCa remains ongoing.

By outlining practical expert recommendations on molecular testing, encouraging broader adoption across centres, and advocating for the inclusion of pathologists in the tumour board (country-dependent), we hope our collective perspectives can support more HCPs in confidently incorporating HRR/*BRCA* testing into routine PCa care.

## Data Availability

No new datasets were generated or analyzed in this study.
