# Supplementary material for: Homologous recombination repair and breast cancer gene testing in prostate cancer: expert perspectives and practical guidance on best practice from sample acquisition to biomarker-informed clinical decision-making
Source: Front Oncol. 2026 Jul 1;16:1874947. doi: 10.3389/fonc.2026.1874947 (PMC13368679; doi:10.3389/fonc.2026.1874947)
Supplement: Supplementary file 1 [file DataSheet1.docx]

**Homologous recombination repair and breast cancer gene testing in prostate cancer: Expert perspectives and practical guidance on best practice from sample acquisition to biomarker-informed clinical decision-making**

| **Guideline** | **Genetic/germline testing*** | **Tumour/somatic testing** |
| --- | --- | --- |
| EAU 2026 | - Advise germline testing in men with multiple family members diagnosed with PCa at age < 60 years or a family member who died from PCa < 60 years (Weak) - Offer germline testing in men with a family history of high-risk germline mutations or a family history of multiple cancers on the same side of the family (Strong) - Offer germline testing to patients with *BRCA* mutations on somatic testing (Strong) | - Somatic testing should be considered for all patients presenting with metastatic PCa and fit enough to consider a PARPi |
|  | - Test mHSPC patients for somatic or germline homologous recombination repair aberrations, since they may qualify for the addition of niraparib to ADT plus abiraterone in patients with M1 disease (Weak) - Offer mCRPC patients somatic and/or germline molecular testing, as well as testing for mismatch repair deficiencies or microsatellite instability, if not done previously (Strong) | |
| ESMO 2026 | - Germline testing is recommended in patients with mHSPC [III, A]   - Panels including cancer susceptibility genes, such as *BRCA1/2*, MMR genes (*MLH1, MSH2, MSH6, PMS2, EPCAM*), and *HOXB13*, can be recommended [III, B]   - Panels including other moderate-risk genes, such as *ATM*, *CHEK2*, and *PALB2*, can also be recommended [III, B]. Other genes can be added based on personal and family history [III, B] - Germline testing can be recommended for patients with mCRPC if it has not been carried out previously [III, B] | - Testing for alterations in *BRCA1* [ESMO Scale for Clinical Actionability of molecular Targets (ESCAT) score: I-A], *BRCA2* [ESCAT score: I-A], *CDK12* [ESCAT score: II-A], and *PALB2* [ESCAT score: II-B] (at a minimum) is recommended for all patients with CRPC, ideally before the first treatment of mCRPC [III, A] - MMR deficiency (dMMR) testing is recommended for all patients with mCRPC after ARPI treatment [III, A; ESCAT score: III-A] |
| NCCN 2026 | - Germline testing is recommended in patients with high-risk localised/very-high-risk localised/regional (node positive)/metastatic PCa, patients with a family history and patients of Ashkenazi Jewish ancestry (2A) - If criteria are met, multigene testing is recommended (2A) - Germline testing should be considered in appropriate individuals where it is likely to impact the PCa treatment and clinical trial options, management of risk of other cancers, and/or potential risk of cancer in family members | - Multigene tumour testing for alterations in HRR genes, including but not limited to *BRCA1/2, ATM, PALB2, FANCA, RAD51D, CHEK2*, and *CDK12*, is recommended in patients with metastatic PCa and can be considered in patents with regional PCa (2A) - Tumour testing for MSI-H or dMMR is recommended in mCRPC and may be considered in patients with regional or castration-sensitive metastatic PCa (2A) - TMB testing is recommended in patients with mCRPC |

**Table 1. Guideline recommendations on molecular testing in prostate cancer (6–8).**
*Genetic counselling is required before and after germline testing (6).

ADT, androgen deprivation therapy; *ATM*, ataxia telangiectasia mutated; *BRCA*, breast cancer gene; *CDK12*, cyclin-dependent kinase 12; *CHEK2*, checkpoint kinase 2; (d)MMR, (deficient) mismatch repair; EAU, European Association of Urology; ESCAT, ESMO Scale for Clinical Actionability of molecular Targets; ESMO, European Society for Medical Oncology; *FANCA*, Fanconi anaemia complementation group A; HRR, homologous recombination repair; M1, metastases stage 1; (m)CRPC, (metastatic) castration-resistant prostate cancer; MSI-H, microsatellite instability-high; NCC, National Comprehensive Cancer Network; PARPi, poly(adenosine diphosphate-ribose) polymerase inhibitor; PCa, prostate cancer; *RAD51D*, RAD51 paralog d; TMB, tumour mutational burden.


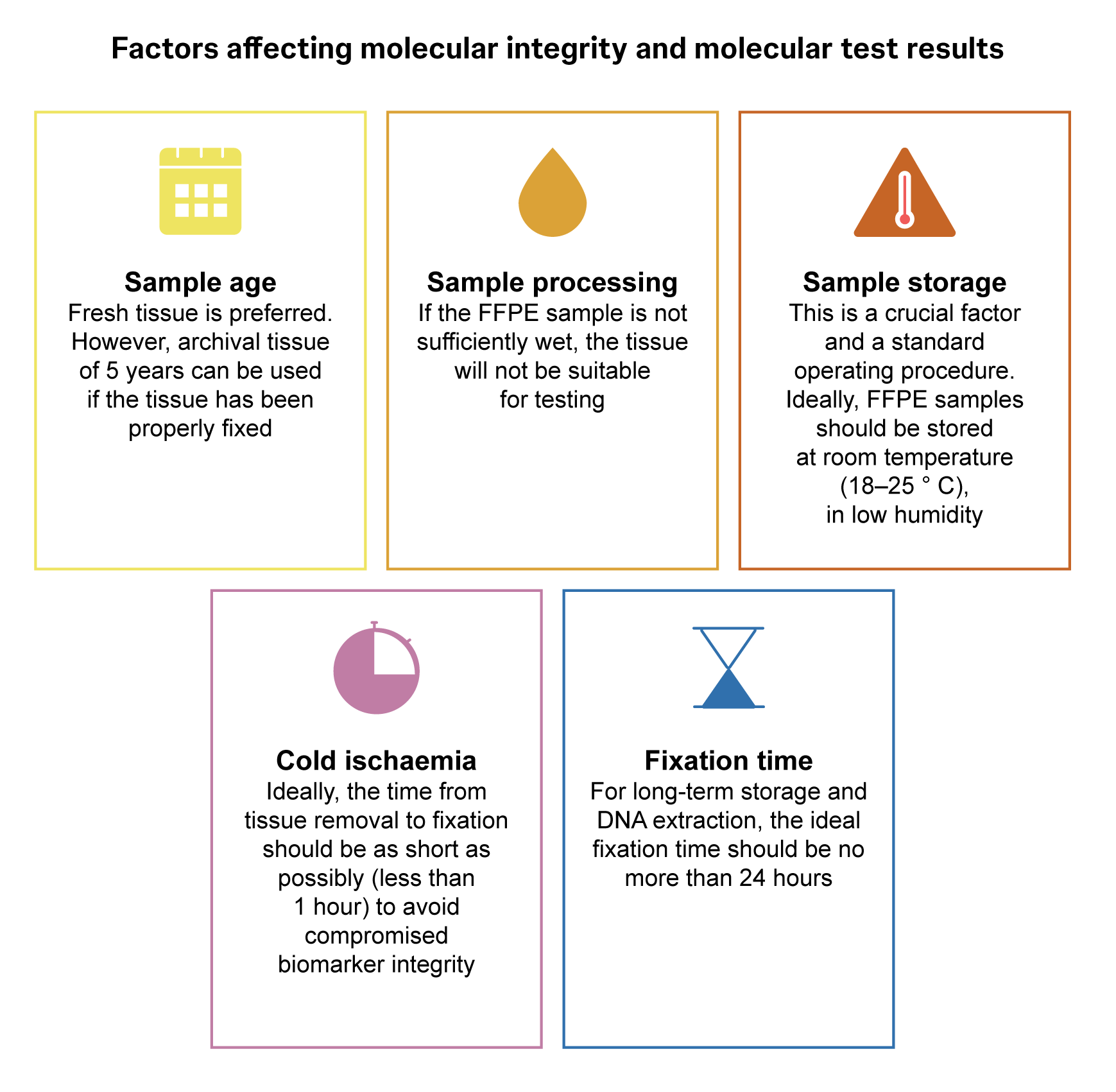

**Figure 1. Factors affecting molecular integrity and molecular test results** (33-36)DNA, deoxyribonucleic acid; FFPE, formalin-fixed paraffin-embedded.


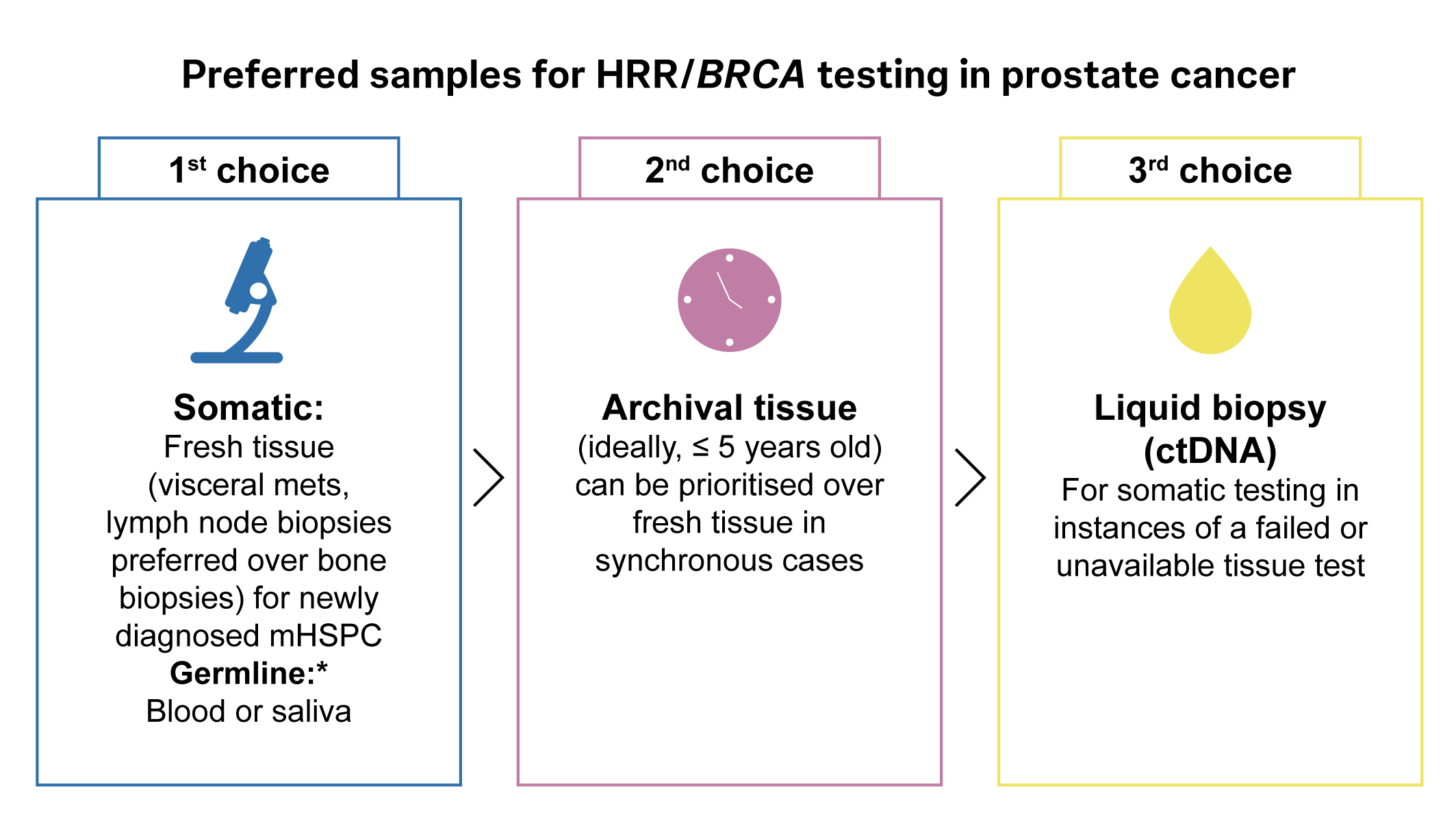


**Figure 2. Samples preferred for HRR/*BRCA* testing.**

*A positive germline test for *BRCA* mutation is enough to classify the patient as *BRCA*-mutation positive.

*BRCA*, breast cancer gene; ctDNA, circulating tumour DNA; HRR, homologous recombination repair; mHSPC, metastatic hormone-sensitive prostate cancer.

| **Common pitfall** | **Possible solution** |
| --- | --- |
| Using a sample that is too old or of poor quality | Request for the most recent tissue block |
| Taking a biopsy from a previously treated/irradiated area or from necrotic tissue | When unavoidable, inform pathologists if the biopsy site has been previously treated or irradiated, so that tissue adequacy can be assessed and results can be interpreted accordingly |
| Using liquid biopsy while the patient is actively receiving treatment (i.e., fewer circulating tumour cells) | Test all patients with metastatic PCa, following this order of sample preference: soft fresh tissue (if available, for newly diagnosed mHSPC) > archival tissue (prioritised in synchronous cases) > bone biopsy > liquid biopsy (as an alternative). The test should preferably be as early as the first diagnosis of mHSPC |
| Over-fixation or under-fixation | Develop checklists or standardised protocols to guide sample preparation |
| Not enough clinical context/information is provided to pathologists | Develop checklists or standardised protocols |
| Metastatic sample is too small for testing | Perform macrodissection to enrich the tumour  Use archival tissue if metastatic tissue is insufficient  Raise awareness for the need to isolate sufficient metastatic tissue for genetic testing  Consider re-biopsy or liquid biopsy |
| Incomplete mutation detection due to gene panels that are too narrow or limited | Use an integrative NGS-based approach (as validated in breast and ovarian cancers) that detects both germline and somatic mutations in HRR genes |
| Failed or suboptimal testing due to insufficient DNA for the chosen sequencing method | Choose an appropriate NGS platform:  Amplicon-based NGS: requires only ~10 ng DNA  Targeted capture-based NGS: requires 30–300 ng DNA |
| Unclear reports. Molecular test results often do not include estimation of tumour fraction/allele frequency/mean tumour coverage, essential for result interpretation | Develop standardised reporting  Reports should include clear guidance on the significance of variants and potential therapeutic implications to avoid unnecessary repeat testing/missed opportunities |
| **Useful information to include in test reports** | |
| Separately report variants of unknown significance (VUS) from treatment eligibility, clearly stating that no evidence supports the targeted therapy benefit | |
| Potential therapeutic implications of test outcomes | |
| Allele frequency | |
| Mean sequencing coverage for tumour DNA | |
| Estimation of tumour content or tumour fraction | |
| Only include mutations with VAF above the validated limit of detection | |
| If the tumour assay is unable to detect chromosomal rearrangements, the report should clearly state this so that patients with a strong family history can be considered for germline testing | |
| Ensure results comply with the International Organization for Standardization 15189 requirements for medical laboratory quality and competence and relevant national guidelines (58) | |
| Classify and rank all identified genomic alterations according to the ESMO Scale for Clinical Actionability of molecular Targets to inform clinical discussions and treatment choice (59) | |

**Table 2. Ways to minimise failure rates and avoid common pitfalls associated with HRR/*BRCA* testing in metastatic prostate cancer and useful information to include in test reports.** Useful information adapted from Gonzalez D, et al. 2021 (35).

DNA, deoxyribonucleic acid; ESMO, European Society for Medical Oncology; HRR, homologous recombination repair; mHSPC, metastatic hormone-sensitive prostate cancer; NGS, next-generation sequencing; PCa, prostate cancer; VAF, variant allele frequency; VUS, variant of unknown significance.


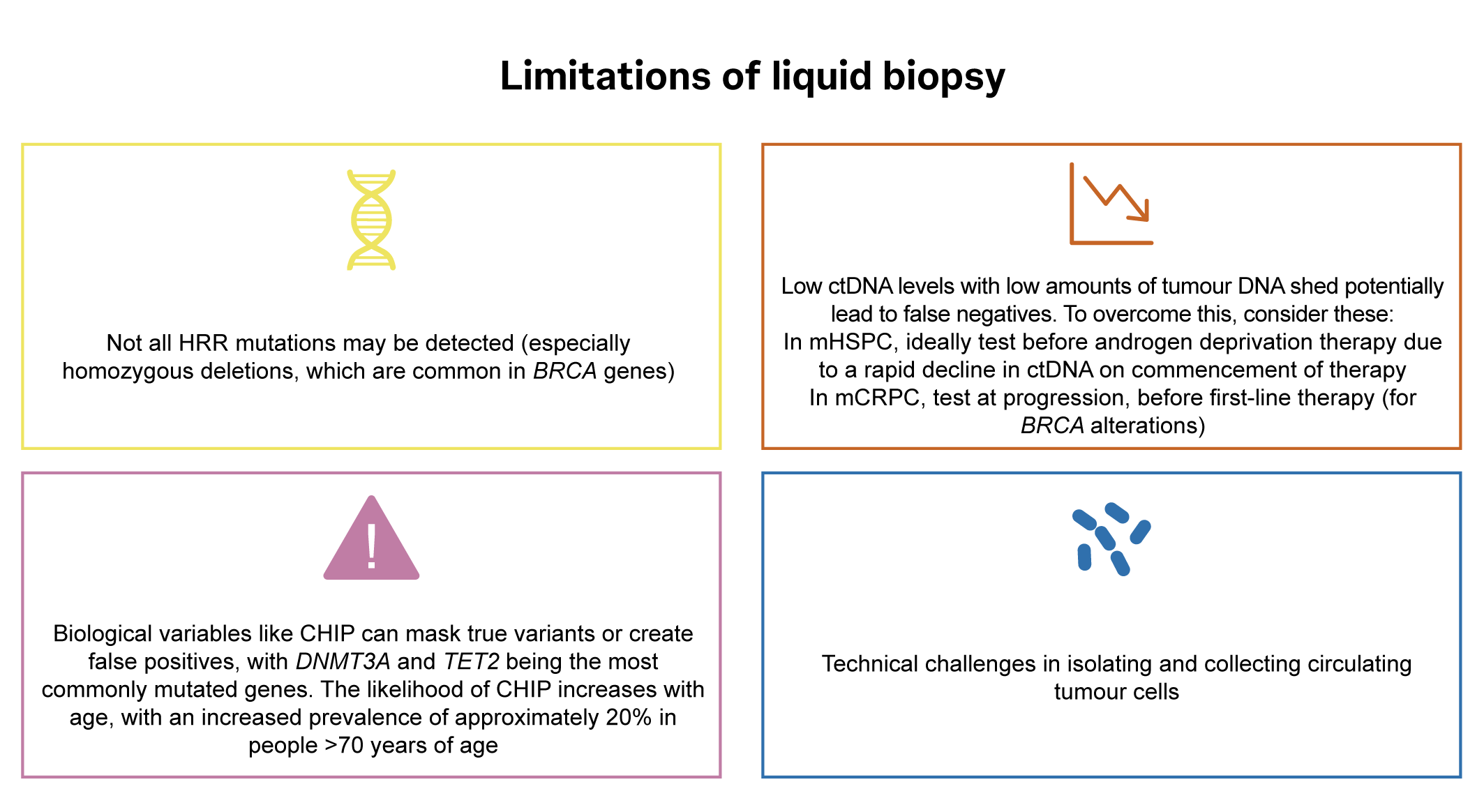
**Figure 3. Limitations of liquid biopsy (45,47-53)**.

*BRCA*, breast cancer gene; CHIP, clonal haematopoiesis of indeterminate potential; ctDNA, circulating tumour deoxyribonucleic acid; *DNMT3A*, DNA methyltransferase 3 alpha; HRR, homologous recombination repair; mCRPC, metastatic castration resistant prostate cancer; mHSPC, metastatic hormone-sensitive prostate cancer; *TET2*, ten–eleven translocation 2.
